# The use of telemedicine with patients in clinical practice: the view of medical psychology

**DOI:** 10.1590/S1516-31802013000100011

**Published:** 2013-02-01

**Authors:** Décio Gilberto Natrielli, Décio Gilberto Natrielli, Mailu Enokibara

**Affiliations:** I MD. Attending Psychiatrist, Irmãs Hospitaleiras do Sagrado Coração de Jesus, São Paulo, Brazil.; II MD. Scientific Coordinator, Multidisciplinary Committee of Medical Psychology, Associação Paulista de Medicina (APM), São Paulo, Brazil.; III MD. Resident, Department of Psychiatry, Centro de Atenção Integrada a Saúde Mental (CAISM), Santa Casa de Misericórdia de São Paulo, São Paulo, Brazil.

After reading the short communication “Initial experience at a university teaching hospital from using telemedicine to promote education through video conferencing” by Pereira et al.^1^ we felt that it would be useful to briefly report on our interest in this field of research, particularly its clinical application and the outcomes for patients attended by physicians. We decided to focus on the use of mobile phones, landline telephones, text messages and even computer software that allows audiovisual communication, and aimed to describe the practical consequences of improving and expanding our care for patients beyond the time of routine ambulatory service.

It is common knowledge that many patients, even with clinicians’ efforts to clarify doubts and provide information about treatments, go to pharmacies and then their homes feeling insecure and anxious about the treatment outcome and the risks of side effects. It might be said that this seems very obvious and that such equipment should be used by all healthcare professionals, although physicians’ opinions may vary from a belief in full availability to “keep in touch” with the patient, to total absence of contact outside the office (albeit with an ethical orientation to refer cases to an emergency room in the event of any side effects or complications).

This letter aims to highlight the phenomenon that all devices used (and defined as telemedicine) are extensions of the medical setting and physicians’ environment. They act as a reference point for patients, sometimes via “magical thoughts” about the doctor’s power to provide or control the expected treatment result. Hence, they lead to different types and polarities of patient behavior: dependence or independence; security or insecurity; trust or mistrust; confidence or low self-esteem; affective or hostile attitudes; anxiety or calmness; depressive or exalted states; and other descriptions of human feelings.

Medical psychology is a field that permeates all physicians’ activities, with emphasis on the psychological aspects of the doctor-patient relationship.^2^ Even without all the technological apparatus available for telemedicine practice, doctors still have the possibility of strengthening the bond between physicians and patients beyond the office environment: sometimes clarifying doubts about side effects or medications, and at other time teaching patients, families or other healthcare professionals how to deal with a specific situation within a certain specialty, by means of a simple telephone or mobile phone call. In fact, this is the principle of telemedicine, which has nowadays evolved to more sophisticated hardware and software. Berg et al. provided a study protocol using a “telephone and text-message based telemedical care concept for patients with mental health disorders”. These authors described several studies that evaluated telemedical concepts: videoconferences, video consultation, telephone consultations and e-mail contacts.^3^

With evolutionary technology at hand, the “side effects” must be taken to be an important warning to all professionals who deal with patients. One possible consequence that needs to be borne in mind is the risk of paradoxical results, given that with all these communication facilities, it cannot be guaranteed that people will really communicate with each other and be closer to one another. In such an eventuality, the paradoxical reality would be “detachment” of some vulnerable people, thereby making them spatially and emotionally isolated. However, this is another issue beyond our interest in this letter, since this point of view does not take into consideration epidemiological studies that use telemedicine but, rather, individual and singular behavioral features.

Our practical application includes modest telemedicine resources, as described above, and we believe that, even with a simple telephone or mobile phone, better care for patients can be provided, through always returning phone calls, answering questions and teaching those who share in caring for others. Expansion of practice to more advanced devices like videoconferencing should be the ideal reference point and, with the current technology available, we are moving in this direction. Perhaps, one day, it will be possible to provide better assistance for families and patients and narrow the gap in communication with other professionals, with the aims of consultation, sharing information and debating and discussing clinical issues.
